# Application of a MRI based index to longitudinal atrophy change in Alzheimer disease, mild cognitive impairment and healthy older individuals in the AddNeuroMed cohort

**DOI:** 10.3389/fnagi.2014.00145

**Published:** 2014-07-14

**Authors:** Carlos Aguilar, J-Sebastian Muehlboeck, Patrizia Mecocci, Bruno Vellas, Magda Tsolaki, Iwona Kloszewska, Hilkka Soininen, Simon Lovestone, Lars-Olof Wahlund, Andrew Simmons, Eric Westman, for the AddNeuroMed Consortium

**Affiliations:** ^1^Division of Clinical Geriatrics, Department of Neurobiology, Care Sciences and Society, Karolinska InstitutetStockholm, Sweden; ^2^Department of Neuroimaging and Department of Old Age Psychiatry, Institute of Psychiatry, King's College LondonLondon, UK; ^3^Institute of Gerontology and Geriatrics, University of PerugiaPerugia, Italy; ^4^INSERM U 558, University of ToulouseToulouse, France; ^5^Department of Classics, Aristotle University of ThessalonikiThessaloniki, Greece; ^6^Department of Old Age Psychiatry and Psychotic Disorders, Medical University of LodzLodz, Poland; ^7^Department of Neurology, University and University Hospital of KuopioFinland; ^8^NIHR Biomedical Research Centre for Mental Health and NIHR Biomedical Research Unit for DementiaLondon, UK

**Keywords:** MRI, Alzheimer disease, MCI, APOE4, multivariate statistics, OPLS

## Abstract

Cross sectional studies of patients at risk of developing Alzheimer disease (AD) have identified several brain regions known to be prone to degeneration suitable as biomarkers, including hippocampal, ventricular, and whole brain volume. The aim of this study was to longitudinally evaluate an index based on morphometric measures derived from MRI data that could be used for classification of AD and healthy control subjects, as well as prediction of conversion from mild cognitive impairment (MCI) to AD. Patients originated from the AddNeuroMed project at baseline (119 AD, 119 MCI, 110 controls (CTL)) and 1-year follow-up (62 AD, 73 MCI, 79 CTL). Data consisted of 3D T1-weighted MR images, demographics, MMSE, ADAS-Cog, CERAD and CDR scores, and APOE e4 status. We computed an index using a multivariate classification model (AD vs. CTL), using orthogonal partial least squares to latent structures (OPLS). Sensitivity, specificity and AUC were determined. Performance of the classifier (AD vs. CTL) was high at baseline (10-fold cross-validation, 84% sensitivity, 91% specificity, 0.93 AUC) and at 1-year follow-up (92% sensitivity, 74% specificity, 0.93 AUC). Predictions of conversion from MCI to AD were good at baseline (77% of MCI converters) and at follow-up (91% of MCI converters). MCI carriers of the APOE e4 allele manifested more atrophy and presented a faster cognitive decline when compared to non-carriers. The derived index demonstrated a steady increase in atrophy over time, yielding higher accuracy in prediction at the time of clinical conversion. Neuropsychological tests appeared less sensitive to changes over time. However, taking the average of the two time points yielded better correlation between the index and cognitive scores as opposed to using cross-sectional data only. Thus, multivariate classification seemed to detect patterns of AD changes before conversion from MCI to AD and including longitudinal information is of great importance.

## Introduction

Alzheimer disease (AD) is one of the most common forms of neurodegenerative disorders characterized by a gradual loss of cognitive functions such as episodic memory. A revision of the National Institute of Neurological and Communicative Disorders and Stroke (NINCDS) and the Alzheimer disease and related Disorders Association (ADRDA) criterion (McKhann et al., 1984) for the diagnosis of AD has been suggested. The new research criterion is still centered on a clinical core of early and significant episodic memory impairment, but also includes at least one abnormal biomarker among magnetic resonance imaging (MRI), positron emission tomography (PET) and cerebrospinal fluid (CSF) (Albert et al., 2011; McKhann et al., 2011; Sperling et al., 2011). Since evidence for use of these biomarkers is growing, it strengthens their role in the diagnosis of AD. MRI provides structural information about the brain and has for many years been widely used for early detection and diagnosis of AD (O'Brien, 2007; Ries et al., 2008; Tondelli et al., 2012). With this technique it is possible to measure both regional (hippocampus/entorhinal cortex) and global (whole brain) atrophy, which are considered sensitive surrogate markers, capable of quantifying the extent of brain degeneration in dementia (Apostolova et al., 2006). However, more atypical patterns of AD have also been observed with different clinical presentations. Brain atrophy patterns characterized by hippocampal volume loss in limbic-predominant cases and increased temporoparietal and frontal atrophy in hippocampal sparing cases, with typical AD cases showing atrophy in all regions (Whitwell et al., 2012). A goal of structural neuroimaging analysis in AD is to identify a pattern of atrophy (regional and/or global) that can help predict further cognitive decline or establish a probable diagnosis. It is possible to obtain multiple volumetric and cortical thickness measures from high resolution MRI by automated segmentation techniques. Multivariate techniques enable utilization of information from different regions of the brain to distinguish between different dementias and patient groups (Teipel et al., 2007; Davatzikos et al., 2008; Klöppel et al., 2008; Vemuri et al., 2008; Magnin et al., 2009; Plant et al., 2010; Westman et al., 2011). Moreover, it has been proposed that structural information can be condensed into an index by using multivariate techniques (Fan et al., 2008; Mattila et al., 2011; Spulber et al., 2013).

We have previously introduced an index derived from a multivariate discrimination model [orthogonal partial least squares to latent structures (OPLS)] (Spulber et al., 2013). The index was characterized based on cross-sectional data from the AddNeuroMed and ADNI cohorts. In the present study we expand the analysis to longitudinal MRI data with the aim to longitudinally evaluate the index. For this purpose; (1) a multivariate classification model was trained using baseline AD and control (CTL) MRI scans; (2) the derived classification model was applied to AD, CTL (1-year follow-up) and MCI individuals (baseline and 1-year follow-up); (3) resulting in an individual index which was used to compute descriptive statistics and perform group analysis; (4) finally, the index annual rate of change was calculated and compared to the cognitive scores' annual rate of change, and the average index was calculated and compared to average cognitive scores for each diagnostic group, and after stratification by atrophy pattern and APOE e4 status.

## Materials and methods

### Subjects and inclusion criteria

**Figure 1:**
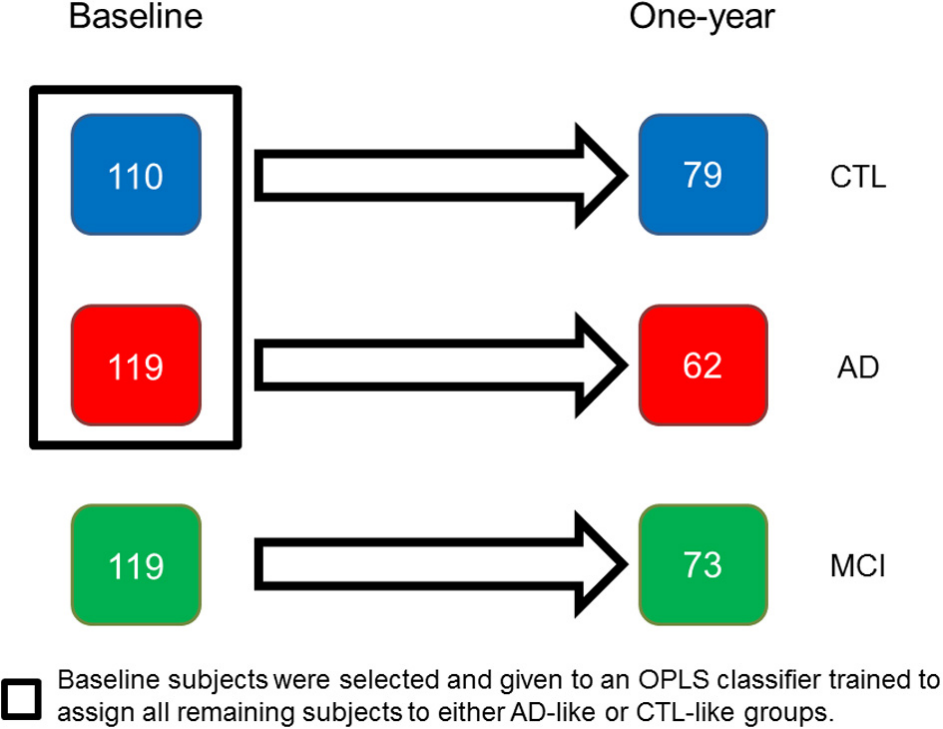
**AddNeuroMed longitudinal population: at baseline 348 individuals were assessed using cognitive and MRI methods, with 214 individuals subsequently followed longitudinally**. AD, Alzheimer disease; MCI, mild cognitive impairment; CTL, healthy control.

All patients originated from the AddNeuroMed project, part of InnoMed (Innovative Medicines in Europe), a European Union programme designed to make drug discovery more efficient. The project is designed to develop and validate novel surrogate markers in AD and includes a human neuroimaging strand (Simmons et al., 2009, 2011) which combines MRI data with other biomarkers and clinical data. Data was collected from six different sites across Europe: University of Kuopio, Finland, University of Perugia, Italy, Aristotle University of Thessaloniki, Greece, King's College London, United Kingdom, University of Lodz, Poland, and University of Toulouse, France. MR images from a total of 119 AD patients, 119 MCI patients, and 110 healthy controls (CTL) were available. Only a subsample of 62 AD patients, 73 MCI patients and 79 CTL individuals were followed longitudinally however. Table 1 provides demographics of the whole and longitudinal datasets, Table 2 summarizes the cognitive scores per diagnostic group, and Figure 1 depicts the disposition of the diagnostic groups. All AD and MCI subjects were recruited from local memory clinics of the six participating sites while the control subjects were recruited from non-related members of the patient's families, caregiver's relatives or social centers for the elderly. Written consent was obtained where the research participant had capacity, and in those cases where dementia compromised capacity then assent from the patient and written consent from a relative, according to local law and process, was obtained. This study was approved by ethical review boards in each participating country. The inclusion and exclusion criteria were as follows. **Alzheimer disease**: *Inclusion criteria*: (1) ADRDA/NINCDS and DSM- IV criteria for probable AD. (2) Mini Mental State Examination score range between 12 and 28. (3) Age 65 years or above. *Exclusion criteria*: (1) Significant neurological or psychiatric illness other than AD. This would exclude patients with vascular dementia or large infarcts, for example. (2) Significant unstable systematic illness or organ failure. All AD subjects had a Clinical Dementia Rating (CDR) scale score of 0.5 or above. **Mild Cognitive Impairment and controls**: *Inclusion criteria*: (1) Mini Mental State Examination score range between 24 and 30. (2) Geriatric Depression Scale score less than or equal to 5. (3) Age 65 years or above. (4) Medication stable. (5) Good general health. *Exclusion criteria*: (1) Meet the DSM- IV criteria for Dementia. (2) Significant neurological or psychiatric illness. (3) Significant unstable systematic illness or organ failure. The distinction between MCI and controls was based on: the subject scores 0 on Clinical Dementia Rating Scale = control. The subject scores 0.5 on Clinical Dementia Rating scale = MCI. Additionally, for the MCI subjects it was preferable that the subject and informant reported occurrence of memory problems. There is an overlap regarding CDR-sum-of-boxes and MMSE scores between AD and MCI patients.

**Table 1:** **Subject demographics at baseline**.

	***N***	**Gender M/F**	**Age**	**Years of education**	**APOE e4 carriers**
**WHOLE AddNeuroMed DATASET^a^**					
AD	119	41/78	75.5 ± 6.0	8.0 ± 4.0	64 [54%]
CTL	110	50/60	73.0 ± 6.5	10.8 ± 4.8	31 [28%]
MCI	119				
MCI-c	22	14/8	72.8 ± 6.4	9.2 ± 4.3	13 [59%]
MCI-nc	97	46/51	74.7 ± 5.4	8.8 ± 4.3	26 [27%]
**AddNeuroMed LONGITUDINAL DATASET^b^**					
AD	62	16/46	74.0 ± 5.6	8.6 ± 4.0	34 [55%]
CTL	79	35/44	74.5 ± 5.5	10.3 ± 4.5	25 [32%]
MCI	73				
MCI-c	13	8/5	71.8 ± 5.7	9.5 ± 4.3	8 [62%]
MCI-nc	60	30/30	75.7 ± 0.7	9.2 ± 4.4	15 [25%]

*Data is presented as mean ± standard deviation. The longitudinal dataset consists of individuals followed longitudinally over 1-year and assessed using cognitive batteries and MRI scans. AD, Alzheimer disease; CTL, healthy control; MCI-c, MCI converters; MCI-nc, MCI non-converters; MCI, Mild cognitive impairment*.

a *Significant differences in Age (AD vs. CTL Mann-Whitney test P = 0.002) and years of education (AD vs. CTL and MCI vs. CTL Mann-Whitney test P < 0.004)*.

b *Significant differences in Age (MCI-c vs. MCI-nc Mann-Whitney test P = 0.04), gender (AD vs. MCI Mann-Whitney test P = 0.009) and years of education (AD vs. CTL Mann-Whitney test P = 0.014)*.

**Table 2:** **Cognitive scores at baseline and 1-year follow-up**.

	**AD**		**MCI**		**CTL**	
	**Baseline**	**1-year**	**Baseline**	**1-year**	**Baseline**	**1-year**
**WHOLE AddNeuroMed DATASET**						
MMSE	20.9 ± 4.8	–	27.1 ± 1.7	–	29.1 ± 1.2	–
CDR-sum	6.5 ± 3.3	–	1.4 ± 0.9	–	0.1 ± 0.2	–
ADAS1	6.6 ± 1.5	–	5.6 ± 1.2	–	3.8 ± 1.5	–
**AddNeuroMed LONGITUDINAL DATASET**						
MMSE	22.2 ± 4.4	21.6 ± 4.9	27.2 ± 1.7	26.3 ± 2.9	29.1 ± 1.2	28.8 ± 0.3
CDR-sum	5.6 ± 3.0	7.2 ± 4.0	1.6 ± 0.9	2.1 ± 1.5	0.1 ± 0.2	0.1 ± 0.4
ADAS1	6.3 ± 1.5	6.5 ± 1.8	5.5 ± 1.2	5.5 ± 1.4	3.9 ± 1.6	3.7 ± 1.5

*Data is presented as mean ± standard deviation. AD, Alzheimer disease; MCI, mild cognitive impairment; CTL, healthy control; MMSE, Mini Mental State Examination; CDR-sum, Clinical Dementia Rating sum of boxes; ADAS1, Alzheimer Disease Assessment Scale*.

At baseline, a health interview was administrated to all participants following a standardized protocol available at http://www.innomed-addneuromed.com and data on demographics, medical history, current health status, medication use and family history were collected. For AD and MCI cases, information on the duration and severity of cognitive decline was obtained from the subjects or informants through a detailed questionnaire: the presence of memory problems, time of their onset, subsequent course, and investigations performed.

For all participants, cognition, behavior, functional status, and global severity were assessed and the MMSE (Folstein et al., 1975) and the Clinical Dementia Rating scale (CDR) (Hughes et al., 1982) were administered. Evaluation of AD included the Hachinski ischemic scale (Hachinski et al., 1975), the AD assessment scale-cognitive subscale (ADAS-Cog) (Rosen et al., 1984), Neuropsychiatric Inventory (Cummings et al., 1994), and the Alzheimer disease Cooperative Study (ADCS)-activities of daily living scale (Galasko et al., 1997). Evaluation of MCI and CTL individuals comprised the Consortium to Establish a Registry for Alzheimer Disease (CERAD) Cognitive Battery (Morris et al., 1989) and the Geriatric Depression Scale (GDS) (Yesavage and Sheikh, 1986). The same procedure was used at 12 month follow-up. MRI findings were not known to clinicians, so as not to influence the clinical diagnosis.

The ADAS-cog and the CERAD battery use the same 10-word recall task. The only difference is that the scoring is inverted. The mean number of words not recalled in the CERAD word list immediate recall task was calculated. The variable obtained was named ADAS1, corresponding to the first subtest of ADAS-Cog. This was performed to obtain comparable measures between groups. CDR global was used for diagnosis as described above, but CDR sum of boxes were used in relation to the index.

### MRI acquisition

Data acquisition for the AddNeuroMed study was designed to be compatible with the Alzheimer Disease Neuroimaging Initiative (ADNI) (Jack et al., 2008). Data was collected from 4 General Electric, 1 Siemens, and 1 Picker MR-scanner. The imaging protocol for both studies included a high resolution sagittal 3D T1-weighted MPRAGE volume (voxel size 1.1 × 1.1 × 1.2 mm^3^) and axial proton density/T2-weighted fast spin echo images. The MPRAGE volume was acquired using a custom pulse sequence specifically designed for the ADNI study to ensure compatibility across scanners. Full brain and skull coverage was required and detailed quality control carried out on all MR images according to the AddNeuroMed quality control procedures (Simmons et al., 2009, 2011).

### Regional volume segmentation and cortical thickness parcellation

FreeSurfer (version 4.5.0) was used for image processing and analysis. The pipeline produces regional cortical thickness and volumetric measures. Cortical reconstruction and volumetric segmentation includes removal of non-brain tissue using a hybrid watershed/surface deformation procedure (Segonne et al., 2004), automated Talairach transformation, segmentation of the subcortical white matter and deep gray matter volumetric structures (including hippocampus, amygdala, caudate, putamen, ventricles) (Fischl et al., 2002, 2004; Segonne et al., 2004), intensity normalization (Sled et al., 1998), tessellation of gray matter and white matter boundary, automated topology correction (Fischl et al., 2001; Ségonne et al., 2007) and deformation following intensity gradients to optimally place the gray/white and gray/CSF borders at the location where the greatest shift in intensity defines the transition to the other tissue class (Dale and Sereno, 1993; Dale et al., 1999; Fischl and Dale, 2000). Once the cortical models are complete, registration to a spherical atlas takes place, which utilizes individual cortical folding patterns to match cortical geometry across subjects (Fischl et al., 1999). This is followed by parcellation of the cerebral cortex into units based on gyral and sulcal structure (Fischl et al., 2004; Desikan et al., 2006). The pipeline generated 68 cortical thickness measures (34 from each hemisphere) and 50 regional volumes. Volumes of white matter hypointensities, optic chiasm, right and left vessel, and right and left choroid plexus were excluded from further analysis. White matter hypointensities were excluded since most subjects were characterized by zero values. Cortical thickness and volumetric measures from the right and left side were averaged. In total 57 MRI measures were used as input variables for multivariate analysis, see Table 3. Using tkmedit, the image processing steps were visually inspected to ensure they had been carried out correctly and corrected for skull-stripping errors and gray/white matter boundary (cases where significant image artifacts or gross brain abnormalities result in segmentation errors were excluded). These segmentation approaches have been used for multivariate classification of Alzheimer's disease and healthy controls (Westman et al., 2010; Mangialasche et al., 2013), neurophysiological image analysis (Liu et al., 2011; Ferreira et al., 2014), imaging-genetic analysis (Liu et al., 2010b; Velayudhan et al., 2013), and biomarker discovery (Thambisetty, 2008; Thambisetty et al., 2010).

**Table 3:** **FreeSurfer variables included in the study**.

**Cortical thickness**	**Subcortical volume**
Banks of superior temporal sulcus	Third ventricle
Caudal anterior cingulate	Fourth ventricle
Caudal middle frontal gyrus	Brainstem
Cuneus cortex	Corpus callosum anterior
Entorhinal cortex	Corpus callosum central
Fusiform gyrus	Corpus callosum midanterior
Inferior parietal cortex	Corpus callosum midposterior
Inferior temporal gyrus	Corpus callosum posterior
Isthmus of cingulate cortex	CSF
Lateral occipital cortex	Accumbens
Lateral orbitofrontal cortex	Amygdala
Lingual gyrus	Caudate
Medial orbitofrontal cortex	Cerebellum cortex
Middle temporal gyrus	Cerebellum white matter
Parahippocampal gyrus	Hippocampus
Paracentral sulcus	Inferior lateral ventricle
Frontal operculum	Putamen
Orbital operculum	Cerebral cortex
Triangular part of inferior frontal gyrus	Cerebral white matter
Pericalcarine cortex	Lateral ventricle
Postcentral gyrus	Pallidum
Posterior cingulate cortex	Thalamus proper
Precentral gyrus	Ventral DC
Precuneus cortex	
Rostral anterior cingulate cortex	
Rostral middle frontal gyrus	
Superior frontal gyrus	
Superior parietal gyrus	
Superior temporal gyrus	
Supramarginal gyrus	
Frontal pole	
Temporal pole	
Transverse temporal cortex	
Insula	

### Data preprocessing

All volumetric measures from each subject were normalized by the subject's intracranial volume (ICV). The ICV was estimated based on an affine transform in Freesurfer, while cortical thickness measures were not normalized (Westman et al., 2013). Preprocessing of each variable was performed using mean centering and unit-variance scaling.

### Orthogonal partial least squares to latent structures

Orthogonal partial least squares to latent structures (OPLS) (Trygg and Wold, 2002; Bylesjö et al., 2006; Wiklund et al., 2008) is a supervised multivariate data analysis method included in the software package SIMCA (Umetrics AB, Umea, Sweden). In OPLS, the information related to class separation is found in the first component of the model, the predictive component. The orthogonal components in the model, if any, relate to variation in the data not connected to class separation. Focusing the information related to class separation on the first component makes data interpretation easier (Wiklund et al., 2008).

Each OPLS model receives a Q^2^(*Y*) value that describes its statistical significance for separating groups. Q^2^(*Y*) values > 0.05 are regarded as statistically significant (Eriksson et al., 2006), where

$$Q^{2}(Y)=1-PRESS/SSY$$

PRESS (predictive residual sum of squares) = ∑ (*y*_*actual*_ − *y*_*predicted*_)^2^ and SSY is the total variation of the *Y* matrix after scaling and mean centering (Eriksson et al., 2006). Q^2^(*Y*) is the fraction of the total variation of the *Y*s (expected class values) that can be predicted by a component according to cross-validation (CV).

### Index: high-dimensional classification from multiple MRI features

An OPLS model was created at baseline for AD vs. CTL (119 AD and 110 CTL). Cortical thickness measures and subcortical volumes were analyzed separately, which means a separate OPLS model is created for each set of variables. The two models are then combined using hierarchical modeling (Westman et al., 2012). The OPLS model creates an index for each individual subject, which tends to be close to 1 for AD patients and 0 for CTL individuals. Index values above 0.5 indicate a more AD-like characteristic while values below 0.5 a more CTL-like characteristic. The AD vs. CTL OPLS model derived from the training data was applied on the longitudinal data of AD, CTL, and MCI individuals. This produced an index reflecting the degree to which the individual's pattern resembled the pattern of AD patients or the pattern of CTL individuals. Although age and education were significantly different between groups, the model did not consider them as confounding variables since multivariate models seemed to be robust to the presence of differences in age and/or years of education because the overall pattern of atrophy is a strong predictor of AD in and of itself (Klöppel et al., 2008; Vemuri et al., 2008; Misra et al., 2009; Aguilar et al., 2013).

### Statistical comparisons

Basic descriptive statics were used to characterize diagnostic groups and stratified according to a specific criterion (e.g., gender or time point). Linear regression was used to illustrate dependences and quantified by computing linear correlation coefficients. Statistical significance was determined by applying repeated One-Way ANOVA and non-parametric matched-paired test. Area under the ROC (receiver operating characteristic) curve (AUC) was calculated using the web-based application JROCFIT (Eng J. ROC analysis: web-based calculator for ROC curves. Baltimore: Johns Hopkins University; www.jrocfit.org).

## Results

### OPLS model creation

The resulting classification model enabled high discrimination between AD and CTL individuals (7-fold CV: 84% sensitivity, 91% specificity, 0.93 AUC). As part of the model, the top 10 most important variables for discrimination were: hippocampus, entorhinal cortex, amygdala, temporal pole, superior temporal gyrus, inferior lateral ventricle, middle temporal gyrus, fusiform gyrus, inferior temporal gyrus, and parahippocampal gyrus (see Table 3 for a list of all variables included in the analysis).

### Index

**Figure 2:**
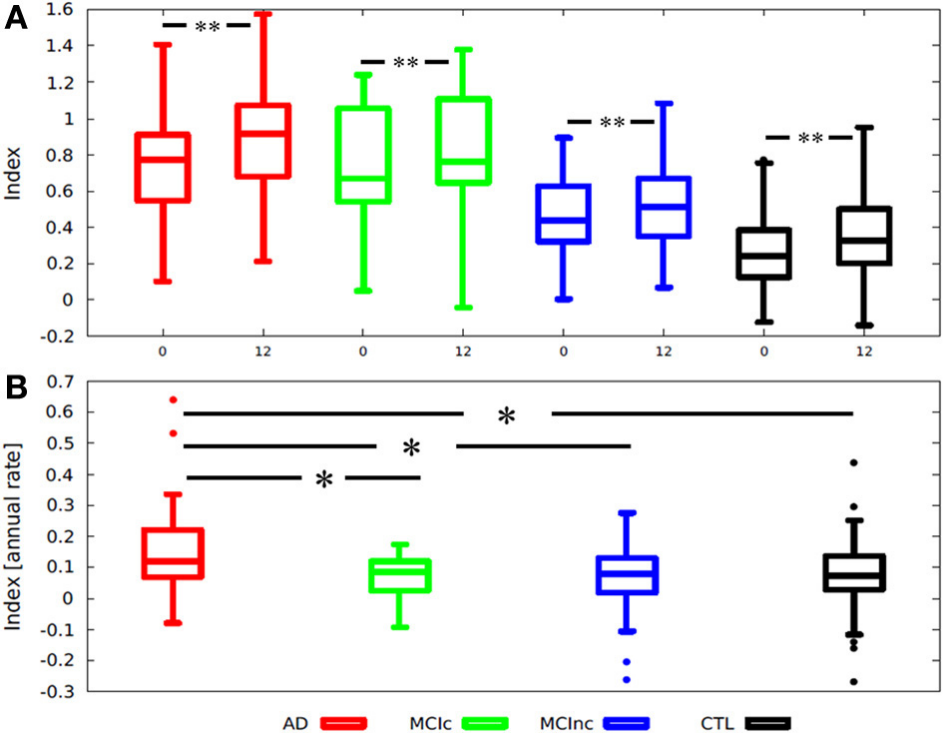
**Box-plots illustrating the distribution of (A) an MRI index according to diagnosis and stratified by time point; (B) Index annual rate of change stratified by diagnosis**. Index annual rate of change = index at 1-year − index at baseline. ^*^One-tailed *T*-test *p* < 0.002; ^**^One-tailed paired *T*-test *p* < 0.003; AD, Alzheimer disease; MCI-c, MCI converter; MCI-nc, MCI non-converter (stable); MCI, mild cognitive impairment; CTL, healthy control.

**Figure 3:**
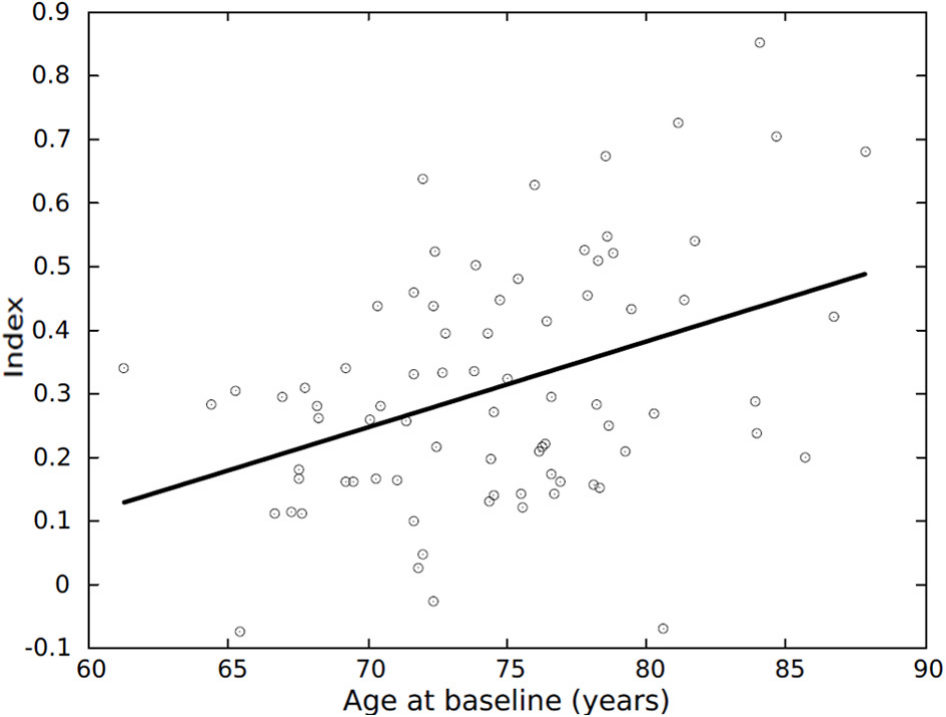
**Average index vs. age, for the 79 CTL individuals part of the longitudinal dataset**. Average index = (index at baseline + index at 1-year)/2. CTL, healthy control.

The index was computed for all individuals in the longitudinal dataset. Figure 2A and Table 4 summarize the distribution of the index per diagnostic group, per time-point; and Figure 2B summarizes the distribution of the index annual rate of change per diagnostic group. Overall, we observed increased atrophy over time, AD patients showed the fastest increase (One-Way ANOVA *P* = 0.044). The index was highest in the AD group, followed by MCI converters (MCI-c), MCI non-converters (MCI-nc), and the CTL group. CTL subjects had values below 0.5. However, in older individuals with normal cognition AD-like values (>0.5) were more frequent (Figure 3).

**Table 4:** **MRI index computed for the longitudinal dataset**.

	**AD**		**MCI**		**CTL**	
	**Baseline**	**1-Year**	**Baseline**	**1-Year**	**Baseline**	**1-Year**
Average	0.70	0.86	0.49	0.56	0.26	0.36
Stdev	0.29	0.31	0.28	0.30	0.19	0.19
Median	0.74	0.91	0.46	0.55	0.22	0.33
Min	0.10	0.21	0.01	−0.05	−0.13	−0.03
Max	1.21	1.48	1.24	1.38	0.73	0.74
Q1	0.52	0.66	0.32	0.33	0.12	0.20
Q3	0.91	1.08	0.65	0.75	0.42	0.53

*Stdev, Standard deviation; Q1, First quartile; Q3, Third quartile; AD, Alzheimer's disease; MCI, mild cognitive impairment; CTL, healthy control; OPLS, orthogonal partial least squares to latent structures*.

**Figure 4:**
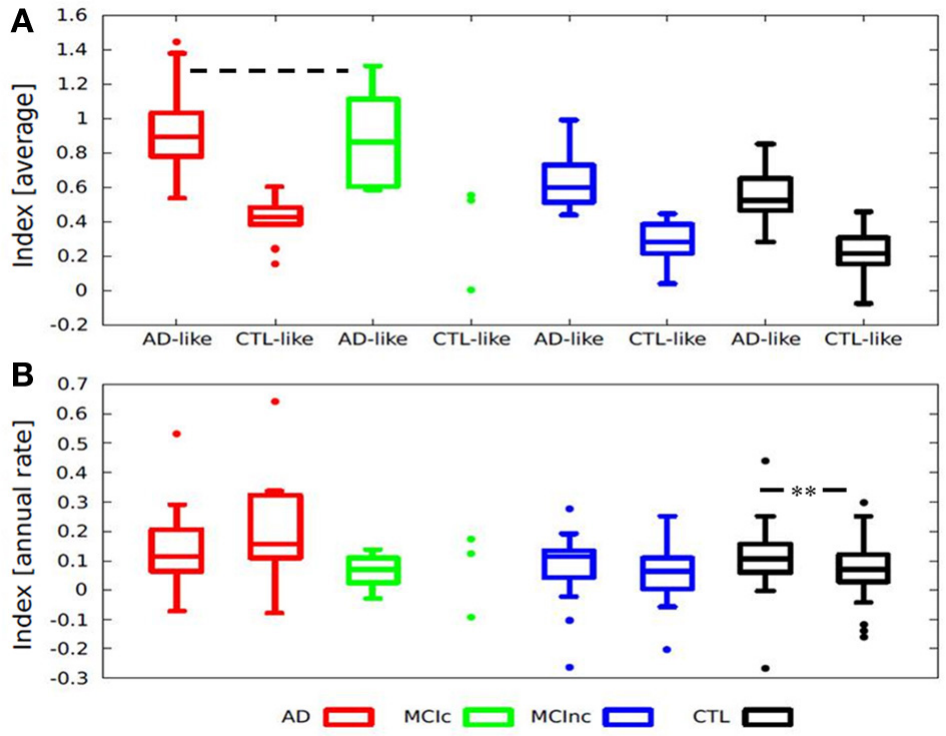
**Box-plots illustrating the distribution of (A) Average index according to diagnosis and stratified by pattern of atrophy, all groups are significantly different except for AD-like AD and AD-like MCI-c (--- Two-sided *T*-test *p* = 0.76). (B)** Index annual rate of change stratified by diagnosis and pattern of atrophy. Index annual rate of change = index at 1-year − index at baseline. ^**^Mann-Whitney *p* = 0.028; Average index = (Index at baseline + index at 1-year)/2. AD, Alzheimer disease; MCI-c, MCI converter; MCI-nc, MCI non-converter (stable); MCI, mild cognitive impairment; CTL, healthy control.

Taking the average index from baseline and follow-up data and stratifying the diagnostic groups according to pattern of atrophy resulted in good separation of AD-like AD patients and CTL-like CTL subjects, as well as AD-like MCI-c and CTL-like MCI-nc patients, and revealed subgroups of subjects with values in between (Figure 4A). Also, for each diagnostic group, having a different pattern of atrophy resulted in a different index of annual rate of change, although non-significant, except for the CTL group (Figure 4B). Those CTL individuals having an AD-like pattern of atrophy had a significantly higher rate of atrophy (Index annual rate of change: AD-like > CTL-like; Mann-Whitney *p* = 0.028).

### OPLS classification and MCI predictions

Sensitivity, specificity and AUC are reported for classification (AD vs. CTL) and prediction of MCI conversion to AD (Table 5).

**Table 5:** **Performance and prediction based on the AD vs. CTL classification model**.

	**AD-like**	**CTL-like**	**Sensitivity (%)**	**Specificity (%)**	**AUC**
TRAINING SET (BASELINE, ***N*** = **348**)^*^					
AD	100	19	84	91	0.93 ± 0.02
CTL	10	100			
LONGITUDINAL DATASET (BASELINE, ***N*** = **214**)^**^					
AD	50	12	81	90	0.90 ± 0.03
CTL	8	71			
MCI-c	11	2	85	65	0.80 ± 0.08
MCI-nc	21	39			
**LONGITUDINAL DATASET (1 YEAR, ***N*** = **214**)**					
AD	57	5	92	75	0.93 ± 0.02
CTL	20	59			
MCI-c	12	1	92	47	0.75 ± 0.09
MCI-nc	32	28			

A training set was used to train a classifier and applied on the longitudinal dataset; see Figure 1 for a description of the datasets. AD, Alzheimer disease; CTL, healthy control. MCI, mild cognitive impairment; MCI-c, MCI converters; MCI-nc, MCI non-converters; AUC, area under the receiver operating characteristic curve.

* *Training model using cross-validation*.

** Values recalculated from the baseline cross-validated model.

Individual classification accuracy was significantly lower (*p* < 0.001) at baseline (7-fold CV: sensitivity 81%, specificity 90%, 0.90 AUC) compared to 1-year follow-up (sensitivity 92%, specificity 75%, 0.93 AUC). Classification accuracy increased due to an increase in sensitivity, but there was a decrease in specificity as well.

MCI individuals were labeled as AD-like or CTL-like based on their individual index. Furthermore, they were stratified as converters (MCI-c) or non-converters (MCI-nc) based on the 1-year follow-up diagnosis. We report the number of MCI-c subjects identified as AD-like. Out of 13 MCI-c subjects 11 were predicted as AD-like from baseline data, while 12 were classified as AD at follow-up.

### Cognitive scores and MRI index

Given that changes in brain structure (e.g., atrophy) should relate to cognitive performance, an exploratory statistical analysis was carried out on the cognitive data available. After stratification by diagnostic group, only CDR-sum showed significantly differences between baseline and 1-year scores for AD (*P* < 0.001) and MCI (*P* < 0.001) patients, reflecting an increased impairment; there were no significant changes in ADAS1 or MMSE.

**Figure 5:**
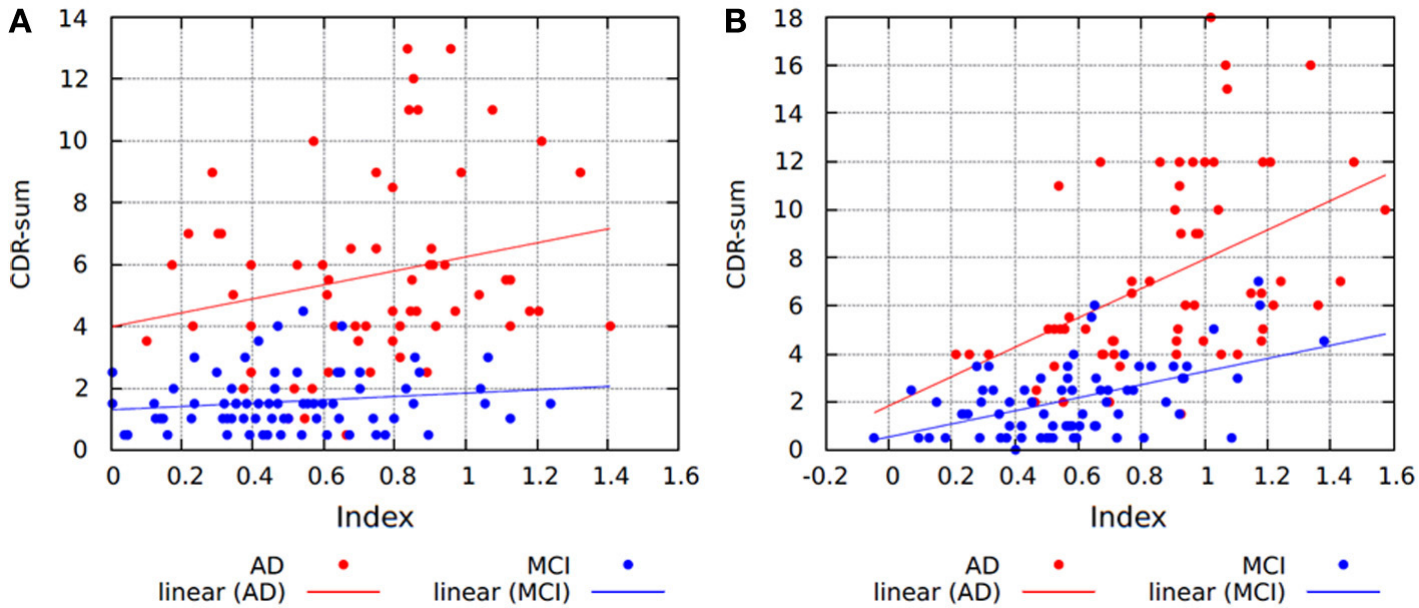
**Scatter plots and regression lines showing the relationship between the index and CDR-sum scores for (A) baseline and; (B) 1-year follow-up data**. AD, Alzheimer disease; MCI, mild cognitive impairment; CDR-sum, clinical dementia rating sum of boxes.

The association between the index and CDR-sum was assessed based on the linear correlation coefficient for each diagnostic group at each time point. At baseline, there was no correlation for MCI patients (Spearman's *r* = 0.220; *P* = 0.093), while a mild non-significant correlation was found for AD patients (Spearman's *r* = 0.29; *P* = 0.063) (Figure 5A). At 1-year follow-up, there was a fair correlation for MCI patients (Spearman's *r* = 0.40; *P* = 0.001), and a good correlation for AD patients (Spearman's *r* = 0.59; *P* < 0.001) (Figure 5B). This suggests that at 1-year follow-up AD and MCI patients became more atrophied and more impaired, and the index and CDR-sum scores became more correlated.

**Figure 6:**
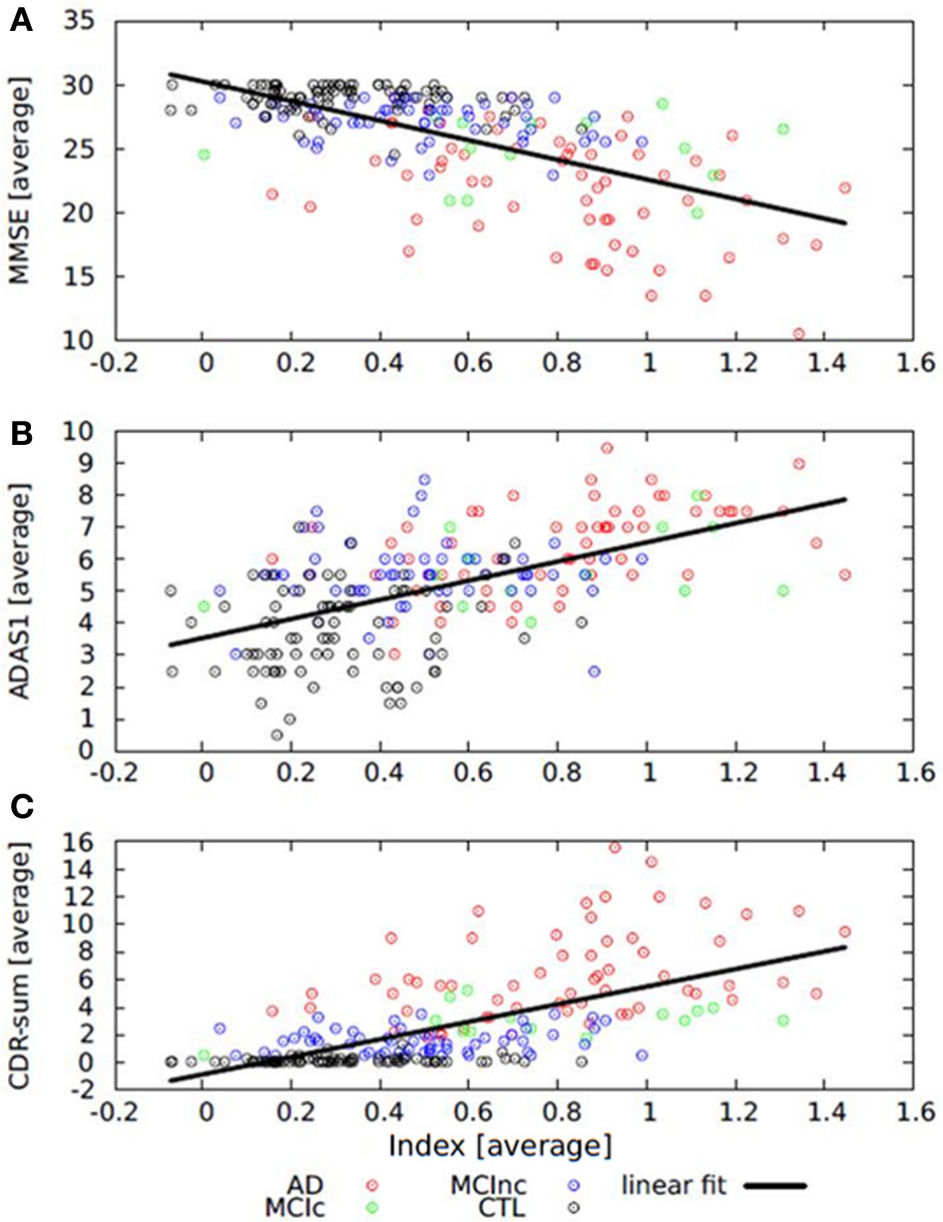
**Scatter plots and regression lines showing the relationship between (A) average index and average MMSE; (B) average index and average ADAS1, and (C) average index and average CDR-sum**. Average index = (average index at baseline + average index at 1-year)/2. MMSE, Mini Mental State Examination; CDR-sum, Clinical Dementia Rating sum of boxes; ADAS1, Alzheimer Disease Assessment Scale.

Since computing the average index from baseline and follow-up data and stratifying by diagnostic groups yielded significant differences for the index analysis, this procedure was also applied on the cognitive measures. Computing the average index and average cognitive scores, there were significant correlations between the index and MMSE (Figure 6A; Spearman's *r* = −0.614, *p* < 0.001), ADAS1 (Figure 6B; Spearman's *r* = 0.562, *p* < 0.001) and CDR-sum (Figure 6C; Spearman's *r* = 0.679, *p* < 0.001). When the analysis was restricted to the AD group, significant correlations were found between the average index and average MMSE (Spearman's *r* = −0.438, *p* < 0.001), average ADAS1 (Spearman's *r* = 0.549, *p* < 0.001) and average CDR-sum (Spearman's *r* = 0.412, *p* = 0.001). In the CTL group there was significant correlation only between the average index and average CDR-sum (Spearman's *r* = 0.236, *p* = 0.036). For the MCI groups there were no significant correlations.

Having a different pattern of atrophy may be reflected in having a different cognitive performance. In particular, there was a faster rate of cognitive decline in AD-like subjects compared to CTL-like in MMSE (for AD Mann-Whitney *p* = 0.035; for CTL Mann-Whitney *p* = 0.05, one-sided *T*-test *p* = 0.03), ADAS1 (for MCI-c Mann-Whitney *p* = 0.013) and CDR-sum (for AD Mann-Whitney *p* < 0.001). Additionally, stratification of CTL individuals by pattern of atrophy resulted in significant correlations between the average index and average MMSE (only AD-like CTL Spearman's *r* = −0.465, *p* = 0.039), average ADAS1 (only AD-like CTL Spearman's *r* = 0.504, *p* = 0.023) and average CDR-sum (only CTL-like CTL Spearman's *r* = 0.351, *p* = 0.006).

### Effect of APOE on the MCI population

**Figure 7:**
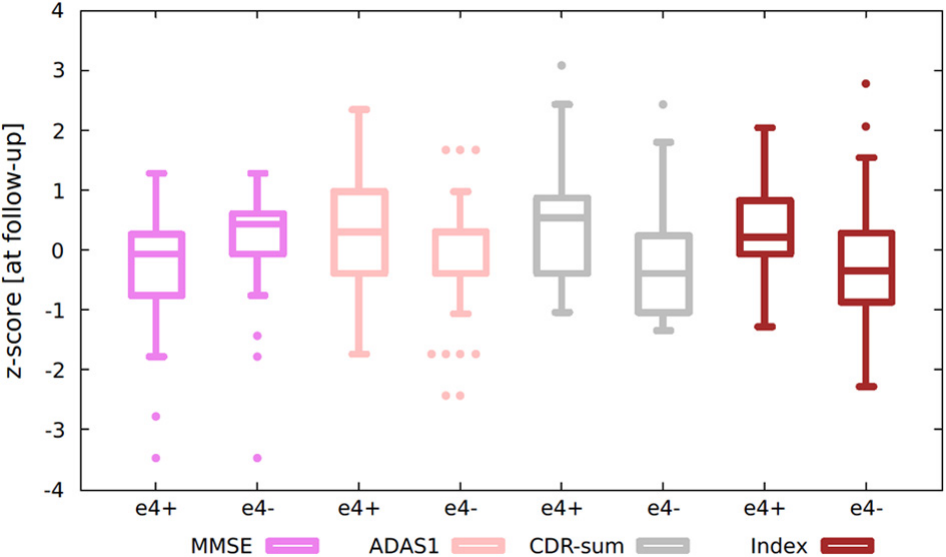
**Box-plots showing stratification by APOE status of (standardized) MCI's index and cognitive scores**. Standardized values were calculated by computing *z*-scores from average data. Average data = (data at baseline + data at 1-year)/2. e4+, APOE e4 carrier; e4−, APOE e4 non-carrier; MCI, mild cognitive impairment; MMSE, Mini Mental State Examination; CDR-sum, clinical dementia rating sum of boxes; ADAS1, Alzheimer Disease assessment scale.

Carrying the APOE e4 allele is the strongest genetic risk factor associated with AD. This was used to stratify individuals as carriers vs. non-carriers and look for an association in cognitive decline consistent with AD pathology. Carriers showed a higher index compared to non-carriers at baseline and follow-up (baseline *P* = 0.041; 1-year *P* = 0.014) indicative of an AD-like atrophy pattern. Moreover, carriers showed an increase in ADAS1 (*P* = 0.017) and CDR-sum (*P* = 0.013) score compared to non-carriers at 1-year follow-up (Figure 7). Therefore, MCI carriers of the APOE e4 allele seemed to be more atrophied and presented a faster cognitive decline compared to non-carriers.

## Discussion

The approach taken in the present study was to extend previous work on a MRI based severity index to include longitudinal MRI data, to investigate changes in the index over time, and its relationship with cognitive performance measures. The approach enabled individual patient classification for identification of AD and CTL subjects with accuracies ranging from 80 to 90%. This classification performance is in agreement with previous results (Aguilar et al., 2013; Spulber et al., 2013). The main regions important for the differentiation of AD vs. CTL were primarily located in the medial temporal lobe as reported in the literature (Davatzikos et al., 2009; Cuingnet et al., 2011; Tondelli et al., 2012; Aguilar et al., 2013; Spulber et al., 2013).

Each individual is predicted by applying the AD vs. CTL classification model which produced an index reflecting the degree to which the individual's MRI pattern of atrophy resembles that of an AD subject or CTL subject, in line with results from similar approaches reported in the literature (Davatzikos et al., 2009; Mattila et al., 2011; Clark et al., 2012).

The number of AD patients identified as AD increased when follow-up data was used. Likewise, the number of MCI patients that would convert to AD identified as AD-like based on their atrophy pattern increased when follow-up data was used. This indicates that the model derived from baseline data captures brain changes, thus convergence to actual diagnosis becomes more accurate with disease progression. However, the number of CTL individuals identified as CTL decreased, and similarly the number of MCI-nc identified as CTL-like decreased. In other words, the index increased indicating that the pattern of brain atrophy resembled more that of an AD patient. Probably due to a natural consequence of aging each individual's index increases over time, and for some individuals, compensatory mechanisms enabled them to cope with brain degeneration and remain cognitively normal. For instance, some older individuals seemed to present a pattern typical of AD pathology even though they had normal cognition, an observation also previously reported (Davatzikos et al., 2009). Further, subjects with a more AD-like pattern at follow-up, may also convert at an even later time-point. There are also important factors such as age to consider which can influence the results, although age provided no significant additional information to the multivariate models (Aguilar et al., 2013). This could be because the multivariate models are robust to the addition of demographic variables. Another possible explanation could be that the overall pattern of atrophy is a strong predictor of AD in itself (Klöppel et al., 2008; Misra et al., 2009; Vemuri et al., 2009), without the need for any additional information such as age. The use of correction methods for age, for example, have also been previously proposed by others (Dukart et al., 2011).

Stratifying each diagnostic group by their pattern of atrophy resulted in significant differences, indicating that a composite index has captured part of the individual variability present in the sample. The subgroup of older CTL with an AD-like pattern of atrophy presented a faster annual rate of atrophy compared to the CTL-like CTL individuals, while the subgroup of AD-like MCI-c and AD-like AD patients showed no significant differences in their average index. These results confirm that MCI patients that will convert to AD (at 1-year follow-up) presented with a pattern of atrophy indistinguishable from that of AD patients. Control subjects presented a heterogeneous distribution of atrophy, some having a more AD-like pattern of atrophy with a faster annual rate of atrophy and faster cognitive decline compared to CTL individuals having a CTL-like pattern of atrophy. Whether this group of healthy individual showing a more AD-like pattern of atrophy represents preclinical AD (Sperling et al., 2013) is difficult to determine and would require a longer follow-up and additional AD biomarkers.

Analysis based on the application of a MRI-based index to track longitudinal atrophy changes suggests that a composite measure consisting of multiple regional brain measures condensed into an index could be used to monitor disease progression. This is in contrast to the observed longitudinal change in cognitive performance over the same period, which resulted in no or mild decrease in cognitive scores. Cognitive tests showed inconclusive proof of further deterioration over 1-year, perhaps because they are based on limited and finite scales insensitive to subtle changes. The index however seemed capable of capturing subtle structural decline thanks to a richer atrophy distribution, thus being able to handle biological and disease heterogeneity. For instance, we observed that CDR-sum score increased at a steeper rate compared to the index. Thus, to overcome this limitation, it has been proposed to create richer cognitive distributions by constructing novel composite scores employing machine learning algorithms, that are able to predict conversion from MCI to AD better than standard cognitive scores (Llano et al., 2011).

The significant association between atrophy and cognition was captured by taking the average index and average cognitive scores, reflecting longitudinal changes rather than cross-sectional measures. More impaired individuals displayed a higher index, therefore reflecting greater severity. Besides severity, when patients were stratified based on patterns of atrophy, having an AD-like pattern of atrophy was associated with a faster rate of cognitive decline when compared to patients having a CTL-like pattern of atrophy. In individuals with preserved cognition stratification by pattern of atrophy revealed associations between cognitive scores and severity of AD. This association may reflect very early mild cognitive decline, since AD-like CTL individuals with higher index values and higher rate of atrophy had an associated cognitive decline. As discussed above, using average values increased the correlation between the index and cognition. Atrophy and cognitive decline are separate but yet tightly connected processes. These processes do not occur simultaneously so by including the longitudinal information, the relationship becomes more evident. Further, the sensitivity of correctly classified AD and MCI-c subjects increases over time, supporting the inclusion of longitudinal information. The increased rate of atrophy observed in AD-like CTL subjects could potentially be used to identify preclinical AD. This again highlights the importance of longitudinal information.

The analysis seems to support the idea that significant atrophy in brain morphology (neurodegeneration) occurs at preclinical stages of dementia and that this does not correlate well with cognitive decline (Burns and Iliffe, 2009; Smith, 2012; Jack et al., 2013), maybe because precise measures of cognitive decline are particularly difficult at this stage, and also because of individual variation due to cognitive reserve and genetic background (Small et al., 2004; Koppel and Goldberg, 2009; Wisdom et al., 2011; Bogdan et al., 2012). In line with these observations, APOE-status seemed to explain some of the individual variability observed in MCI individuals, carriers of the APOE e4 allele seemed not only more impaired, but also more atrophied when compared to non-carriers, suggesting that the APOE e4 allele (Corder et al., 1993, 1995; Lambert and Amouyel, 2011) could modulate the degree of phenotypic expression even in cases of mild cognitive decline (Liu et al., 2010a; Sun et al., 2012). Some of the individual variation in the MCI group could be partially explained by stratifying the sample into carriers and non-carriers. While for AD and CTL individuals there was no significant association with APOE e4 status, possibly indicating that cognitively normal individuals seemed to be well preserved irrespectively of their APOE status (Ferencz et al., 2013; Khan et al., 2014), and AD patients would show a high degree of cognitive decline and atrophy independent of their APOE status (Aguilar et al., 2013). The effect of APOE status has also been investigated in animal models of AD showing that the combination of genetic and lifestyle factors are important (Maioli et al., 2012).

Multivariate methods such as OPLS provide a way to separate groups based on the simultaneous contribution of several input morphometric variables. Such multivariate methods offer the advantage of requiring few statistical comparisons to obtain good generalization and good estimates of confidence (OPLS and SVM). The clinical value of such techniques can be demonstrated by producing models with high predictive value that help identify individuals at risk of cognitive decline atypical of normal aging and closer to dementia patients, either independently or in combination with other established methods (Hampel et al., 2010; Tondelli et al., 2012).

## Study limitations

Only 1-year longitudinal data was available for analysis, and this short follow-up is a limitation that needs to be considered when interpreting the results. For instance, it is difficult to establish the correct final diagnosis for MCI-nc patients due to the short follow-up, some of these patients may convert to AD in the future, while some may revert to cognitively normal. It is also difficult to determine whether CTL subjects are not already in a preclinical stage of AD, or whether this is part of normal variation in healthy aging; however, in addition to a longer follow-up, the inclusion of other biomarkers would be needed to establish whether a cognitively normal subject is already in a preclinical stage. Finally, none of the AD patients is known to have changed diagnosis to another dementia type; however information is only available up to 1-year follow-up.

## Conclusion

MRI as a measure of neurodegeneration shows dynamic and independent atrophy in several brain areas. As an alternative to monitoring individual patterns of brain degeneration from multiple individual regions, the temporal evolution of brain degeneration can be characterized by a condensed index. This approach offers the advantage of facilitating the integration of other clinical biomarkers into a simplified and integrated dynamic model of individual progression. Based on a descriptive statistical analysis, it was possible to identify correlates of cognition and severity, as well as identify potential modulators of individual variation such as genetic background. This interaction can be positively correlated with AD pathology and explains some inter individual variability (APOE e4 allele carriers). However, even if it was possible to observe an increase in the severity level over time, neither the knowledge of baseline severity nor its rate of change could predict future rate of cognitive decline. Great variation was observed: patients having high index and preserved cognitive status, as well as patients having poor cognitive performance and low index. Stratification by pattern of atrophy reduced part of the variation observed and adding the longitudinal information markedly increased the relationship between the index and cognition. Of both research and clinical interest, a longer follow-up will be necessary to determine whether CTL individuals with unusually high index values are at greater risk for memory impairment and eventual conversion to AD. The higher rate of change in the index in AD-like control subjects represents an interesting group for further study to determine if they harbor pre-clinical AD. This is relevant both in the context of clinical trials and early clinical diagnosis. Further studies should focus on individual tracking of disease development using cognitive, structural, functional, and metabolic biomarkers and test whether the proposed index remains valid as a surrogate of neurodegeneration, or more broadly as a proxy of brain integrity.

### Conflict of interest statement

The authors declare that the research was conducted in the absence of any commercial or financial relationships that could be construed as a potential conflict of interest.
